# The effect of temperature on cardiovascular disease hospital admissions among elderly people in Thai Nguyen Province, Vietnam

**DOI:** 10.3402/gha.v7.23649

**Published:** 2014-12-08

**Authors:** Pham Ngan Giang, Do Van Dung, Kim Bao Giang, Hac Van Vinhc, Joacim Rocklöv

**Affiliations:** 1 Administration of Science technology and Training, Ministry of Health, Hanoi, Vietnam; 2 Department of Medical Statistics, University of Medicine and Pharmacy at Ho Chi Minh city, Ho Chi Minh City, Vietnam; 3 Institute for Preventive Medicine and Public Health, Hanoi Medical University, Hanoi, Vietnam; 4 Faculty of Public Health, University of Medicine and Pharmacy, Thai Nguyen, Vietnam; 5 Epidemiology and Global Health, Department of Public Health and Clinical Medicine, Umeå University, Umeå, Sweden

**Keywords:** hospital admission, cardiovascular diseases, temperature, distributed lag non-linear model

## Abstract

**Background:**

Projected increases in weather variability due to climate change will have severe consequences on human health, increasing mortality, and disease rates. Among these, cardiovascular diseases (CVD), highly prevalent among the elderly, have been shown to be sensitive to extreme temperatures and heat waves.

**Objectives:**

This study aimed to find out the relationship between daily temperature (and other weather parameters) and daily CVD hospital admissions among the elderly population in Thai Nguyen province, a northern province of Vietnam.

**Methods:**

Retrospective data of CVD cases were obtained from a data base of four hospitals in Thai Nguyen province for a period of 5 years from 2008 to 2012. CVD hospital admissions were aggregated by day and merged with daily weather data from this period. Distributed lag non-linear model (DLNM) was used to derive specific estimates of the effect of weather parameters on CVD hospital admissions of up to 30 days, adjusted for time trends using b-splines, day of the week, and public holidays.

**Results:**

This study shows that the average point of minimum CVD admissions was at 26°C. Above and below this threshold, the cumulative CVD admission risk over 30 lag days tended to increase with both lower and higher temperatures. The cold effect was found to occur 4–15 days following exposure, peaking at a week's delay. The cumulative effect of cold exposure on CVD admissions was statistically significant with a relative risk of 1.12 (95% confidence interval: 1.01–1.25) for 1°C decrease below the threshold. The cumulative effect of hot temperature on CVD admissions was found to be non-significant and was estimated to be at a relative risk of 1.17 (95% confidence interval: 0.90–1.52) for 1°C increase in the temperature. No significant association was found between CVD admissions and the other weather variables.

**Conclusion:**

Exposure to cold temperature is associated with increasing CVD admission risk among the elderly population.

The changing of the climate system is well documented and complex. It includes changes in the variability and average temperature, humidity, precipitation, and sea level. Global climate change is part of human-induced global environmental changes caused by greenhouse emissions aggravated by deforestation and ocean saturation (1).

The impact of climate change is likely to be crucial in water resources, agriculture, forestry, fishery, energy, transportation, and health sectors (1–4). Indirect health impacts are also caused by changing microbial ecology (1, 5), which increases risk of infectious diseases such as malaria and dengue fever (6, 7), and diarrheal diseases (8). Direct human health impacts include increased mortality and morbidity as results of heat waves, extreme weather events, and temperature-enhanced levels of urban air pollutants. The impacts of heat and cold temperatures have been documented in international studies (9, 10) and studies carried on in North America (11–13), Europe (14, 15), Asia (16, 17), and Australia (18–20), with a significant effect of heat waves on mortality and morbidity being described. In developed countries, there have been several studies on the specific effects of extreme temperatures on stroke and cardiovascular events (11, 21–23). There have been pathophysiologic explanations of these effects. Low temperature causes blood vessels to narrow, which increases blood pressure and the risk of stroke and other cardiovascular events (24) while high temperature causes blood vessels to dilate, which increases cardiac output and risk of decompensate heart failure (25, 26). Both low and high temperatures put stress on the cardiovascular system, especially among the elderly with limited adaptive responses, and increase the risk of coronary heart disease (27, 28).

Impacts of climate change will likely affect poor nations (3, 4) and poor people the most (25). However, in developing countries, studies on direct impacts of climate change on human health, which help to develop policy to mitigate the problems, are scanty. We have not found any quantitative publication on the impact of extreme temperatures on cardiovascular morbidity in Vietnam.

Vietnam has historically been a poor country, but has lately experienced a rapid economic, demographic, and epidemiological transition (29). Vietnam has been traditionally a country with mainly communicable diseases, but recently a mixed pattern of communicable (including classical and emerging infectious diseases) and non-communicable diseases (e.g. cardiovascular disease, cancer, diabetes, mental illness) are characterizing the current epidemiological health profile of the population (30). Geographically, Vietnam is situated in a tropical and monsoon area of the Southeast Asia peninsula. It lies in the tropic inner belt of the Northern hemisphere and is profoundly impacted by the Sea of East Vietnam. Sun elevation angle at noon time is lesser and length of day light is more varied, thus the radiation balance is lower and more varied in the North of Vietnam than in the South of Vietnam. Therefore, the temperature in winter is lower and the seasonal variation in temperature is higher in the North than in the South (31). For example, in Ho Chi Minh city, the average high temperature in May is 33°C and the average low temperature in December is 22°C, whereas in Thai Nguyen, the average high temperature in June is 32°C and the average low temperature in January is 11°C (32). Annual average precipitation commonly is within the range of 1,400–2,400 mm (31).

In this study, we aim to find out the relationship between weather variables such as hours of sunshine, relative humidity, temperature, evaporation, rainfall, and CVD hospital admissions among the elderly in Thai Nguyen province by analyzing the medical records of daily hospitalizations over the period 2008 to 2012.

## Methods

This study was conducted in Thai Nguyen, a northern province of Vietnam with a total population of 1,131,300. Thai Nguyen includes a city as the center of the province and eight rural districts. The population density is 318 km^2^. This density is higher than that of the whole country which is 259. The number of males per 100 females was 97.6. Nine percent of the population was aged 60 or above (33).

Daily weather data were obtained for the Thai Nguyen province over the period 2008 to 2012 from Vietnam National Climate and Weather Center, Hanoi. Selected weather data include daily high temperature, daily low temperature, daily average temperature (degrees celsius), daily average air pressure (mbar), daily average humidity (%), daily evaporation (mm), daily precipitation (mm), and daily sunshine time (hours). The daily weather parameters were averaged over all weather stations in the province to produce a geographically representative exposure measure.

All hospital admissions due to cardiovascular diseases (CVD) over the 5-year period were included in the study. The hospital admissions were collected from four national and provincial hospitals of Thai Nguyen. There is one national hospital in this province named Thai Nguyen National General Hospital (TNGH), and three provincial hospitals referred to as hospital A, hospital C, and hospital of Corporation of Iron and Steel. Hospital admission data were extracted from computerized patient database by Departments of Planning and Informative Center. Data include patients’ age, sex, date of admission, and final diagnosis (as made by attending physicians at discharge). CVD cases included acute myocardial infarction, angina pectoris, congestive heart failure, hypertension, and stroke. All CVD hospital admissions were recorded, and from this all 18,975 elderly (>60 years) patient cases were used in data analysis. In our study, we defined elderly cases as people aged 60 or above. This definition is consistent with that stipulated in The Law of Elderly of Vietnam (34).

Statistical analysis was done using R software version 3.0.1. Descriptive statistics for the weather variables and number of CVD admissions are presented in Tables 1 and 2. Spearman's correlation coefficients was used for exploring the monotonic relation between daily admissions and weather variables. Pearson correlation coefficients between the weather variables was used for interpreting if daily admissions are associated with two or more weather variables. Distributed lag non-linear models (DLNMs) were used to estimate the association between CVD hospital admissions and lags of weather variables, adjusted for time trends using natural cubic splines, day of the week, and public holidays with the R package dlnm (35, 36). To account for possible delayed associations, we examined the impact of weather up to 30 days before the admissions. To derive specific estimates of heat and cold slopes, the DLNM was applied with either a ‘V’- or ‘U’-shaped piecewise linear exposure–response relationship. This relationship involved specifying a single or double threshold for temperature and then estimating a log-linear change in risk of admissions above (or below) the hot (or cold) threshold(s).

**Table 1:** Description of daily weather and CVD hospitalized cases during 2008–2012 in Thai Nguyen province

Variables	Mean	SD	5%	50%	95%
Minimum temperature (°C)	21.0	5.3	10.8	22.6	27.4
Average temperature (°C)	23.6	5.5	13.0	25.1	30.4
Maximum temperature (°C)	27.5	6.2	15.6	28.8	35.2
Average humidity (%)	93.4	5.9	81	95	98
Evaporation (mm)	3.13	1.50	0.8	3.1	5.7
Precipitation (mm)	4.7	13.7	0.0	0	28.9
Sunshine time	3.53	3.54	0.0	2.5	9.8
Air pressure (mb)	1006.8	6.8	996.7	1006.4	1018.9
No. of daily CVD admissions	19.4	9.4	6		35
No. of daily elderly CVD admissions	10.4	5.9	2		21

**Table 2:** Percentage and number () of CVD hospital admissions and number of elderly CVD hospital admissions during the years, 2008–2012

Year	Elderly (*N*=18,975)	Total (*N*=35,353)
2008	16.4% (3,111)	16.9% (5,981)
2009	18.1% (3,437)	19.8% (7,017)
2010	19.0% (3,604)	18.6% (6,583)
2011	23.8% (4,519)	22.5% (7,946)
2012	22.7% (4,304)	22.1% (7,826)

## Results

**Fig. 1:**
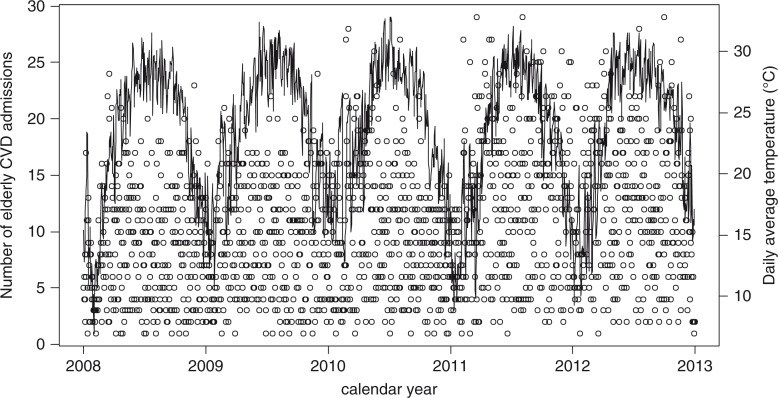
Daily average temperature (line) and number of daily elderly admissions (hollow circles).

Figure 1 presents the elderly CVD admissions (hollow circles) and daily average temperature (solid line) during the 5-year period and shows the seasonal variation of daily average temperature and the erratic variation of elderly CVD admissions. Therefore, the relationship between these two variables is not clear in this graph.

Using Pearson's correlation coefficients, the daily average temperature was strongly inversely correlated with daily average air pressure (*r*=−0.8561, *p*<0.001), and was moderately or weakly positively correlated with daily sunshine hours, daily average humidity, daily evaporation, and daily precipitation with correlation coefficients amounting to 0.4948, 0.2538, 0.2314, 0.1360, respectively (*p*<0.001 for all four above correlations coefficients). Spearman's rank correlation coefficients were used to measure the crude relationship of each pair of temperature, air pressure, evaporation, humidity, precipitation, and hospital admitted cases allowing for potential non-linearities. The correlation coefficients are presented in Table 3 and show a positive correlation of the temperature and negative correlation of daily average air pressure to the elderly CVD admissions. All of these correlations were significant with p-values less than 0.001. Daily evaporation, daily average humidity, and daily precipitation did not show a significant correlation with elderly CVD admissions.

**Table 3:** Spearman's rank correlation between the numbers of elderly CVD admissions and daily weather variability in Thai Nguyen province

	Spearman's rank correlation of elderly CVD cases
Minimum temperature	0.1043*
Average temperature	0.0918*
Maximum temperature	0.0863*
Average air pressure	−0.0810*
Evaporation	0.0157
Average humidity	0.0084
Precipitation	−0.0017

* *p*<0.001.

**Fig. 2:**
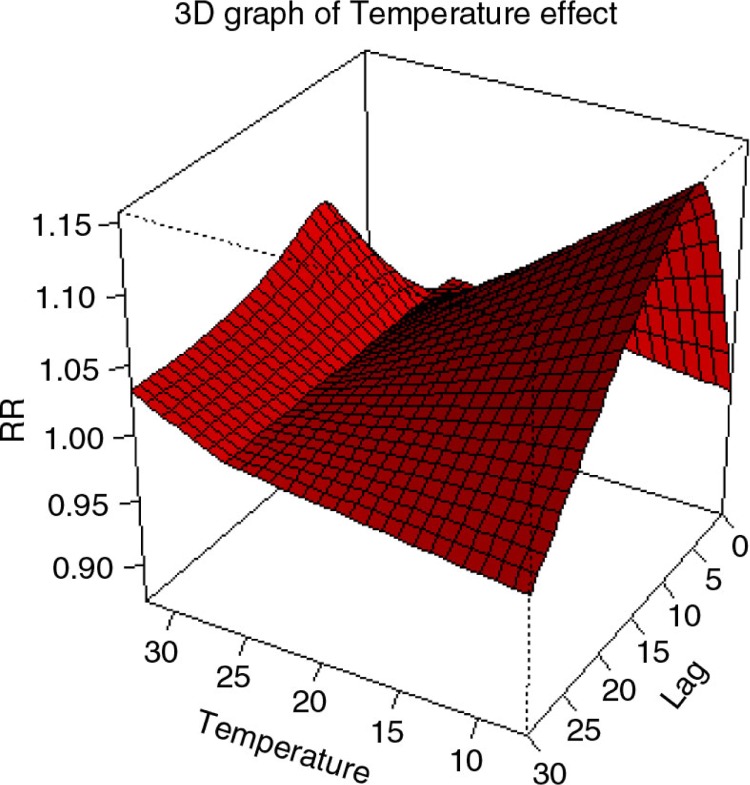
The estimated covariate adjusted effect (relative risk, RR) of daily average temperature on elderly cardiovascular admissions (adjusted to time trends with natural cubic splines, day of the week, and public holidays). The graph shows that the average point of minimum cardiovascular diseases (CVD) admissions was at 26°C. Above and below this temperature, cumulative CVD admission risk over 30 lag days tended to increase.

By using a DLNM with a linear threshold model for temperature, it was found that the exposure–response relationship for CVD admissions was described by a ‘V’ shape with a threshold temperature (of minimum CVD admissions) of 26°C. This relationship was adjusted for other time-varying factors (Fig. 2).

**Fig. 3:**
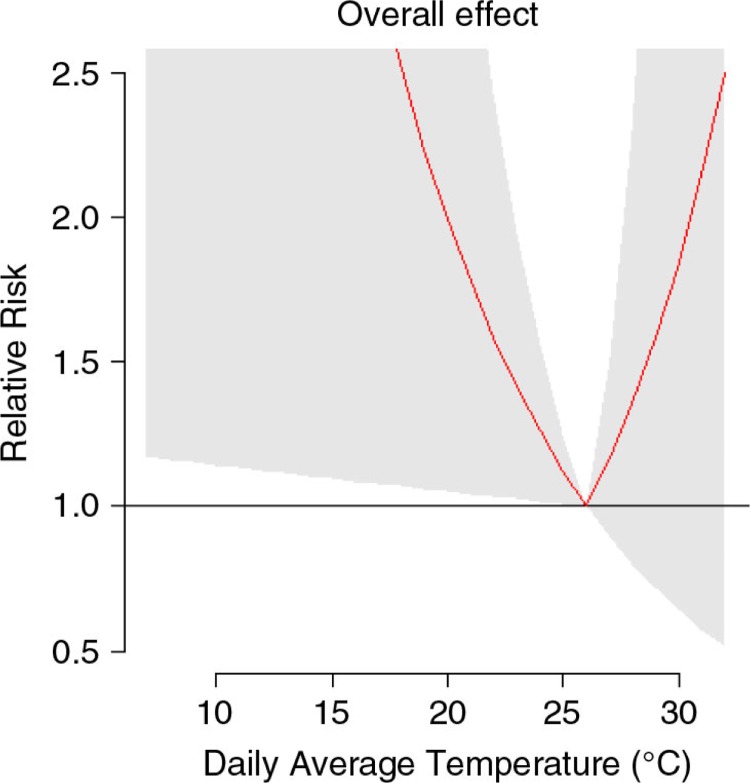
The 30 days’ cumulative relative risk of cardiovascular diseases admission at different daily average temperature. Reference at 26°C. The 95% confident intervals are reported as shaded areas.

The cold effect was found significant while the heat effect was found to be non-significant. The overall effect of cold exposure on CVD admissions over lag 0–30 was estimated at a relative risk of 1.12 (95% confident interval: 1.01–1.25) for 1°C decrease below the threshold. The overall effect of heat exposure on CVD admissions was estimated at a non-significant relative risk of 1.17 (95% confidence interval: 0.90–1.52) for 1°C increase above the threshold (Fig. 3).

**Fig. 4:**
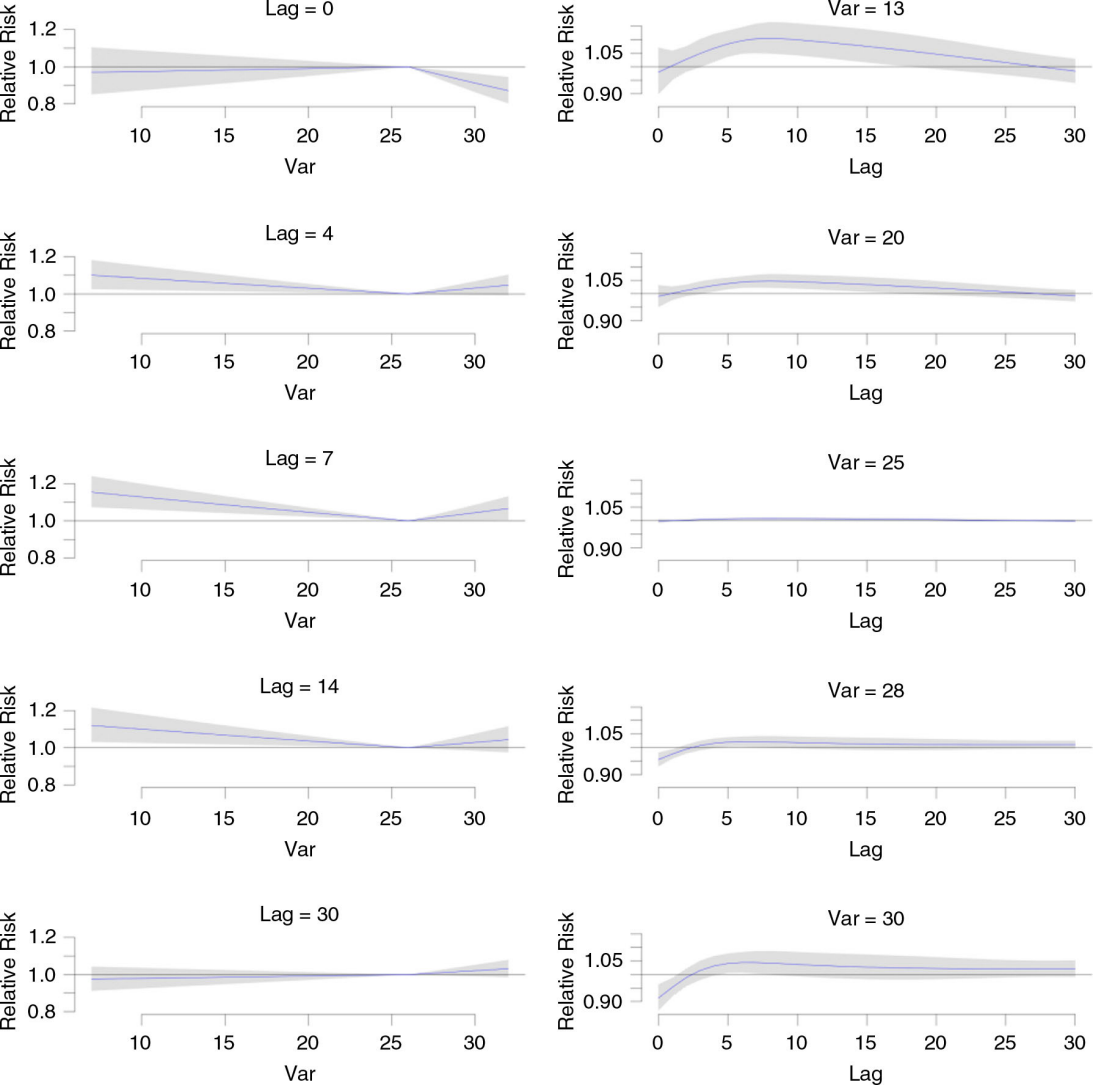
Relative risk (RR) of cardiovascular diseases (CVD) admissions by temperature, which is denoted as Var, and lag using cross-basis smoothing. Left from top: RR by temperature at selected lags 0, 4, 7, 14, and 30°C. Right from top: RR by lag at selected temperatures 13, 20, 28, and 30°C. Risk of CVD admission is scaled to be relative to that at 26°C, the temperature that is sustained so that the CVD admission would be the lowest. Reference at 26°C. The 95% confident intervals are reported as shaded areas.

In Figure 4, the left graphs from top present the relative risk by temperature at selected lags 0, 4, 7, 14, and 30 (the relationship for all lags modeled are presented in Fig. 2). Low temperature did not have a clear effect at lag 0 but significantly increased CVD admission risk at lag 4, lag 7 (most significant), and lag 14, whereas high temperature reduced CVD admission risk at lag 0 and increased the risk of CVD admission at lag 4, lag 7, and lag 14 (but not significant). The effects of both low temperature and high temperature on CVD admissions became negligible at lag 30.

In Figure 4, the right graphs from top present the relative risk by lag at selected temperatures 13, 20, 28, and 30°C. From these graphs, one can see that the effect of low temperature varies by lag time. Cold effect was found to generally occur 4–15 days following exposure, peaking at a week's delay. However, it is clear that overall, low temperatures increase the risk of CVD. The effect of the more extreme temperature (13°C) was more pronounced on CVD admissions than that of less extreme temperature (20°C). The effect of temperature at 25°C on CVD admissions is almost identical to that at 26°C, which is the reference temperature. The effect of temperature of 30°C which is higher than the reference temperature reduced the risk of CVD admissions at lag 0 and increased the risk of CVD admissions from lag 5 to lag 10. However, the overall effect of high temperature is not as pronounced as that of low temperature. The right graphs of Fig. 4 also show that the effects of both low temperature and high temperature on CVD admissions became negligible at lag 30.

Using DLNM, we could not find any association of daily sunshine time, precipitation, or humidity on the elderly CVD admissions (results not shown).

We observed a significant effect of day of the week and public holidays in the elderly CVD admissions. During public holidays, the elderly CVD admissions is reduced by 50%. Weekday patterns revealed that compared to Friday, the admissions on Saturday and Sunday decreased but the admissions on Monday, Tuesday, Wednesday, and Thursday increased.

## Discussions

The goal of this study was to estimate the effects of the weather parameter during the years 2008 to 2012 on elderly CVD hospital admissions in the Thai Nguyen province. This study shows that the average point of minimum CVD admissions was at 26°C. Above and below this threshold, cumulative CVD admission risk over 30 lag days tended to increase with both lower and higher temperatures. Cold effect was found to generally occur 4–15 days following exposure, peaking at a week's delay. The cumulative effect of cold exposure on CVD admission was statistically significant at a relative risk of 1.12 (95% confidence interval: 1.01–1.25) for every 1°C decrease below the threshold of 26°C. The cumulative effect of hot temperature on CVD admission was found to be non-significant and estimating a relative risk of 1.17 (95% confidence interval: 0.90–1.52) for 1°C increase in temperature. No significant association was found between CVD admissions and the other weather variables.

The effect of low temperature on CVD events can be explained by pathophysiology and have been well documented by several studies. At low temperature, the blood vessels become narrow and the blood pressure increase. Cold temperature could lead to thrombosis, and physical activity during cold weather can increase the risk of stable angina and acute coronary syndrome (11, 24). Several studies have also shown that cold temperature increases the risk of CVD events (9, 10, 20, 24, 36). However, the effect of low temperature on CVD admissions found in this study occurs at higher temperature thresholds and are of greater magnitude than the effect found in other studies. This supports the hypothesis that cold effect in a warmer climate, where people usually do not have good houses to protect them from the cold weather, appears to be severe than that in a colder climate (10, 12). The cold effect found in this study generally occurred 4–15 days following exposure, peaking at a week's delay, and is consistent with the results of delayed cold effect found in other studies (17, 36, 37).

In this study, we could not find a significant cumulative heat effect on CVD admission as in some other studies (11, 22). These results are, however, derived for admission and the situation may look different for mortality. For admissions, we observed a potentially large cumulative estimate amounting to 17% increase in mortality per degree increase in heat exposure on warm days. The less strong signal of high temperature on elderly in Thai Nguyen province suggests that the adaptation of people living in warmer climates, through physical adaptation, special housing characteristics or behavioral patterns, may make them less affected by effects of warm temperature. This finding is consistent with the finding from a study based on populations in a hot desert climate (21), and in general when comparing cities with a warmer climate to cities with a cooler climate it appears the heat association is weaker in climatologically warmer areas (9). Another explanation is that, in this study, hypertension is the most common cause of CVD admissions, and cold temperatures increase the blood pressure, whereas the effect of hot temperatures reduces the blood pressure.

Although the cumulative effect of high temperature increases the risk of CVD admissions, one intriguing result in this study is that high temperatures reduced the CVD admissions at lag 0. This reduction counters studies on the effects of hot temperature on mortality (12, 18, 28, 36) and on hospital admission (11), where the effect of increasing risk starts at lag 0. Therefore, this result has to be confirmed or refuted by more studies carried out in developing, tropical countries. However, this may be explained by the fact that a high proportion of CVD admissions are for hypertensive disorders, whereas at high temperatures, the blood pressure will be reduced by decreased sympathetic tone and by dilated blood vessels (38, 39). Non-significant increase in hospital admissions at lag 5 may be due to the harvesting effect (25). The results also indicate a presumed behavioral adaptation to the prevailing weather conditions with respect to health-seeking behavior. Likewise, higher mortality rates in Europe during extreme heat waves are suggested to be partly due to people avoiding the bad weather and staying indoors instead of seeking medical care (2, 40). It is important to study mortality rates in relationship to admissions to examine if the decrease in admissions further increases the associated mortality rates.

In conclusion, the relationship between daily average temperature and elderly CVD admissions found in this study can be described as a ‘V’ shape with an optimal temperature (the average point of minimum CVD admissions) of 26°C, which is higher than the threshold found in studies in temperate climate. This pattern is consistent with the adaptation hypothesis.
